# Rationale and design of the multinational observational study assessing insulin use: the MOSAIc study

**DOI:** 10.1186/1472-6823-12-20

**Published:** 2012-09-21

**Authors:** Jennifer M Polinski, Bradley H Curtis, John D Seeger, Niteesh K Choudhry, Anthony Zagar, William H Shrank

**Affiliations:** 1 Division of Pharmacoepidemiology and Pharmacoeconomics, Brigham and Women’s Hospital, 1620 Tremont Street, Suite 3030, Boston, MA, USA; 2 Harvard Medical School, Boston, MA, USA; 3 Eli Lilly and Company, Indianapolis, IN, USA

**Keywords:** Type 2 diabetes, Insulin, Treatment, Health outcomes, Epidemiology

## Abstract

**Background:**

Although consensus guidelines recommend insulin progression among patients with type 2 diabetes (T2DM) who fail to meet glycemic targets over time, many fewer patients are progressed than may benefit. We describe the rationale and design of the MOSAIc (Multinational Observational Study Assessing Insulin use) study, a multinational observational cohort study to identify patient-, physician, and health care environment-based factors associated with insulin progression for patients with T2DM in real-world practice.

**Methods/design:**

We will enroll 4,500 patients with T2DM taking initial insulin therapy for ≥3 months across 175 physician practice sites in 18 countries. Extensive demographic, clinical, and psychosocial data at the patient and physician level and practice site characteristics will be collected at baseline and regular intervals during a 24-month follow-up period. We will use a multivariable logistic regression model to identify predictors of insulin progression and highlight potential opportunities for health behavior intervention to improve insulin progression rates. Secondary outcomes include evaluating factors associated with glycemic control, hypoglycemia, and treatment adherence among patients who do and do not progress beyond their initial insulin therapy and exploring geographic heterogeneity in treatment.

**Discussion:**

Practice site and patient recruitment began in 2011 and baseline data will be available in late 2012. The MOSAIC study’s longitudinal observational design as well as the breadth and depth of data will be used to explore and quantify predictors of insulin progression and to identify potential opportunities for health behavior intervention in order to improve T2DM treatment and clinical outcomes.

## Background

Both developed and developing countries face a growing epidemic of type 2 diabetes (T2DM). Over 3 million (5.2%) deaths per year are attributable to diabetes [1], making it the fifth leading cause of death worldwide [2]. The prevalence of T2DM is expected to reach 329 million cases globally by 2030, driven by urbanization, aging of the population, and obesity [3]. Treatment and related complications exact a substantial economic toll [4]. The World Health Organization estimates that in the period 2006–2015, China will lose $558 billion in foregone national income due to heart disease, stroke and diabetes [1]. For governments struggling to maximize citizens’ health with limited and often dwindling economic resources, T2DM represents a public health problem of outsized proportions.

### Need for T2DM treatment intensification over time

Because T2DM is a progressive chronic disease, an increasingly intensive treatment strategy is needed to achieve glycemic control. At diagnosis, evidence-based algorithms recommend dietary and activity modifications as well as initiation of metformin [5]. When this treatment strategy, with or without oral anti-diabetic medications, fails to provide adequate glycemic control, insulin initiation is recommended [5,6]. Progression of insulin therapy, defined as switching from basal insulin to premixed insulin, adding bolus doses, and/or increasing the frequency of dosing, is recommended when patients fail to achieve recognized HbA1c targets, which vary between 6.5%-7.5% [5-7]. The widely-used practice guidelines detailed in the American Diabetes Association and the European Association for the Study of Diabetes consensus statement recommends progression if HbA1c levels remain ≥7% after 3 months of basal insulin therapy [5]. However, insulin progression does not occur as often as clinically indicated, with an estimated 75% of patients on insulin monotherapy not achieving their HbA1c targets [8]. Another survey of patient records from academic medical centers found that <50% of eligible patients had therapy progression corresponding to their diabetes status [9], leaving many patients at risk for diabetes complications. Clearly, a disconnect between evidence-based treatment algorithms for insulin progression and their application in real-world settings exists.

### Documented barriers to insulin initiation, but a paucity of data regarding insulin progression

Most existing research has described barriers to insulin initiation and adherence. The cross-sectional, multinational DAWN (Diabetes Attitudes Wishes and Needs) study examined patients’ and healthcare professionals’ attitudes regarding insulin initiation [10]. Patient barriers included: perceived failure in diabetes management; feelings of social stigma and pain; less lifestyle flexibility; injection fears; and erroneous beliefs that insulin is addictive or toxic [11,12]. Health providers reported a reluctance to initiate insulin due to the time and effort burden to teach insulin administration, titrate dosing, and monitor glucose; perceived risks to patients (weight gain, hypoglycemia, worsening of comorbid conditions); and perceived lack of patient adherence to and/or cognitive ability to manage insulin regimens [11,13]. A second cross-sectional study documented health resource and environment-related barriers to insulin initiation, including lack of insurance and access to insulin products/regimens and primary and auxiliary providers [14]. While these studies identified important barriers to insulin initiation, their cross-sectional nature did not allow for an examination of which barriers meaningfully affected clinical outcomes, nor did the studies examine barriers to insulin progression.

Several studies suggest that barriers to insulin progression may be distinct from those for insulin initiation and that their effect on clinical outcomes may differ [15-17]. However, their cross-sectional design limits inferences that can be drawn. There are also few data as to whether treatment guidelines and algorithms for insulin progression are being followed outside of clinical trials. Finally, little is known about patients’ outcomes in routine clinical practice preceding or following insulin progression. Longitudinal, observational studies in real-world settings are needed to identify barriers to insulin progression as well as to quantitatively assess the association between lack of progression and T2DM outcomes.

### Objective

The MOSAIc (Multinational Observational Study Assessing Insulin use: understanding the challenges associated with progression of therapy) study is a multi-national, non-interventional, prospective observational cohort study. Using the Anderson model of health behavior [14,15] framework, the primary objective of MOSAIc is to identify patient, physician, and health care environment factors that influence insulin progression among patients with T2DM and to quantify the relationships between these factors and long-term clinical outcomes. Additional objectives include the evaluation of factors associated with optimal glycemic control, hypoglycemia, and treatment adherence among patients with T2DM who do and do not progress beyond their initial insulin therapy. MOSAIc will also explore geographic heterogeneity with respect to T2DM treatment, insulin delivery modality (pen, syringe, pump), insulin progression, and glycemic control, including the challenges of insulin use among patients who practice fasting for religious and/or cultural reasons [16]. We will examine patient-physician communication regarding diabetes treatment, patients’ diabetes knowledge and self-care behaviors and their impact on glycemic control, and patients’ health-related quality of life as predicted by T2DM knowledge, attitudes, behaviors, and treatment. Comparisons among insulin regimens and their association with cardiovascular health outcomes, hypoglycemia, health care utilization and costs will also be studied. Secondary research objectives are summarized in Table 1.

**Table 1:** Proposed secondary research objectives


**Patient, physician and health environment factors associated with:**
• Glycemic control
• Hypoglycemia
• Treatment adherence
• Diabetes-related health care utilization
• Diabetes-related health costs
**Geographic heterogeneity in:**
• Insulin progression rates
• Insulin treatment modality (pen, syringe, pump)
• Glycemic control
• Patterns of T2DM treatment
**Challenges associated with insulin use:**
• Fasting for religious/cultural reasons
• Health-related quality of life
• Diabetes self-care
• Diabetes knowledge
**Patient-provider communication and impact on:**
• Insulin progression
• Glycemic control
• Hypoglycemia
• Diabetes self-care
• Diabetes knowledge
• Treatment adherence
**Impact of insulin progression on:**
• Cardiovascular outcomes
• Hypoglycemia
• Health-related quality of life
• Diabetes distress
• Diabetes self-care
• Health care resource utilization
• Health care costs

## Methods/design

A total of 175 physician practice sites across 18 countries (Abu Dhabi, Argentina, Brazil, Canada, China, Germany, India, Israel, Italy, Japan, Mexico, Russia, Saudi Arabia, South Korea, Spain, Turkey, United Kingdom, United States including Puerto Rico) will be invited to participate (Table 2). Participating countries were chosen based on geographic region, population aged 20–79, and the T2DM prevalence in each region and country [17]. We will recruit two types of sites, those where physicians practice primary care, internal or general medicine (general medicine sites), and those where physicians practice endocrinology or diabetology (specialty sites). The recruitment goal for each site type reflects the country-specific prevalence of each practice type [18,19] as well as country-specific approaches to T2DM insulin treatment; in some countries, primary care providers manage insulin use, while in others, insulin management is the purview of specialists [20,21]. Both types of sites are approached for participation based on previous research experience with the contract research organization performing recruitment (Quintiles) and/or with Lilly and treatment of at least 4 patients with T2DM per day. Efforts will be made to vary both practice location (urban/rural) and size and type of practice (academic/stand-alone). Using this purposive sampling strategy, our goal is to recruit a heterogeneous population of patients with T2DM using insulin that reflects the underlying population with T2DM in each participating country. Each practice site receives remuneration for the burden associated with data collection and entry. There is no remuneration or incentive for the number of patients enrolled or for the number of patients progressed. At present, practice sites are being actively recruited in 13 of 18 countries, with recruitment efforts planned in the remaining 5 countries in summer 2012. The practice site recruitment rate varies between 25% (Japan and China) and 40% (e.g., U.S., Mexico, Saudi Arabia) at present, with the primary reasons for non-participation being conduct of competing studies, lack of interest in observational research, lack of personnel, perception that remuneration for observational research is too low, and concerns regarding the number of questionnaires. A total of 125 sites are recruiting patients at this time.

**Table 2:** Purposive practice site recruitment strategy

**Region**	**Regional prevalence of diabetes٭**	**Country**	**Total population age 20-79 (in thousands)**	**Country-specific prevalence of diabetes٭**	**Total number of sites**	**Specialty sites**	**General medicine sites**
Southeast Asia	8.1%	China	968,975	9.0%	11	2	9
		India	737,003	9.2%	26	10	16
		Japan	95,341	7.9%	7	4	3
		South Korea	36,204	7.7%	6	4	2
					50	20	30
Europe	10%	Germany	62,810	5.5%	8	2	6
		Italy	45,637	5.3%	6	1	5
		Russia	109,167	10.0%	12	10	2
		Spain	34,896	6.5%	6	1	5
		United Kingdom	44,813	5.4%	6	1	5
					38	15	23
North America	12.1%	Canada	25,141	8.7%	6	1	5
		United States	216,805	9.6%	30	6	24
		Puerto Rico	2,547	13.3%	4	2	2
					40	9	31
Middle East/North Africa	10.8%	Abu Dhabi	6,107	19.2%	6	9	31
		Israel	4,708	7.6%	6	1	5
		Saudi Arabia	17,023	20.0%	6	3	3
		Turkey	47,322	8.1%	11	2	9
					29	7	22
South/Central America	7.8%	Argentina	26,265	5.7%	6	5	1
		Brazil	127,995	10.4%	6	1	5
		Mexico	69,324	15.9%	6	2	4
					18	8	10
		**Totals**			**175**	**59**	**116**

*Age standardized. Obtained from the International Diabetes Federation Diabetes Atlas [17].

At each study site, patients with T2DM who present during the normal course of care and who meet four criteria will be invited to enroll: 1) age ≥18; 2) taking any commercially-available insulin therapy other than intensive basal-bolus insulin therapy (i.e., basal + 3 prandial injections) from any manufacturer for ≥3 months with or without any combination of approved non-insulin anti-diabetic medications; 3) are not simultaneously participating in a study that includes an investigational drug or procedure; and 4) are proficient in the country’s primary language such that they will be able to complete self-report questionnaires. Based on sample size calculations described below, we will enroll 4,500 patients across all sites. The study has received ethics approval in all 18 countries. All patients will complete informed consent forms approved by their country-specific institutional review boards (Additional file 1). The study’s analytic plan has been approved by the Brigham and Women’s Hospital Institutional Review Board.

### Study design and data collection

Upon enrollment, patients’ naturalistic diabetes care and outcomes will be followed prospectively for 24 months. There are no additional treatments, visits, or laboratory collections required beyond those occurring within the course of normal care. A patient may discontinue study participation at any time. Data collection occurs during an initial baseline visit and during four subsequent prospective visit windows (within ±3 months) at 6, 12, 18, and 24 months. Baseline clinical information will be assessed retrospectively from the medical record and includes T2DM diagnosis date, treatment and complications, and T2DM medication history. Diabetes-related health care resource utilization (physician visits, hospitalizations, and auxiliary provider visits: diabetes educators, ophthalmologists, podiatrists, cardiologists, dietitians, and nephrologists), most recent recorded laboratory and vital sign values, and other comorbidities will be assessed at the baseline visit for the period 6 months prior and again at subsequent visits for the period between visits. Other non-anti-diabetic, concurrent medications and demographics will be assessed at the baseline visit. Extensive information on patients’ diabetes- and insulin-related knowledge, attitudes, and behaviors; hypoglycemia and fasting; general health behaviors; patient-provider relationships; and perceived physical and psychological well-being and social status will be collected during visits using validated self-report questionnaires developed for patients with a 6^th^ grade literacy level (Table 3). Patients who have difficulty completing questionnaires may receive assistance as long as responses are their own. Many of the questionnaires are part of the ENSEMBLE MDS, a 68-item battery of instruments designed to explore treatment response heterogeneity regardless of disease state [22]. For example, the ENSEMBLE MDS includes the EQ-5D, which measure general quality of life [23]. Other questionnaires are diabetes- or insulin-specific [25-29]. The full battery of questionnaires takes an average 35–40 minutes to complete. Patients do not receive remuneration for study enrollment nor for questionnaire completion.

**Table 3:** Patient self-reported measures and frequency of collection in the 24 month study period

	**Baseline visit**	**12 month visit**	**24 month visit**	**Any visit at which a patient is progressed**	**Any visit at which a patient discontinues the study**
**Diabetes- and insulin-related knowledge, attitudes, and behaviors**					
Blood glucose monitoring practices	X				
Brief Diabetes Knowledge Test [25]	X		X		X
Diabetes Distress Scale [26]	X		X	X	X
Experience with Insulin Therapy Questionnaire [27]	X		X		X
Hypoglycemia and Fasting Survey (designed by the study team)	X	X		X	X
Insulin Cost Questionnaire (designed by the study team)	X	X	X	X	X
Insulin Specific Adherence Questionnaire (designed by the study team)	X	X	X	X	X
Summary of Diabetes Self-Care Activities [28]	X	X		X	X
**Health status profile (all components part of ENSEMBLE MDS) [**[22]					
Depression diagnosis question	X			X	
Barratt Simplified Measure of Social Status Education question	X			X	
Barratt Simplified Measure of Social Status Occupational Status question	X			X	
MacArthur Income Questionnaire	X			X	
Perceived Social Support Scale	X			X	
Perceived Stress Scale	X			X	
Psychological Health Questionnaire	X			X	
Self-reported health questions (EQ-5D) [23]	X			X	
Total Illness Burden Index				X	
Subjective Social Economic Status				X	
**General health behaviors**					
Smoking and alcohol use	X				
Participation in physical exercise	X				
**Health care access, patient-provider relationship**					
Insurance to pay for prescription medications question	X				
Interpersonal Processes of Care Survey [29]	X		X	X	
Physician: treatment preferences and goals					

Because physicians also play an important role in insulin progression, each physician will complete self-report questionnaires regarding his/her demographics and overall T2DM treatment beliefs and prescribing preferences, including type of device/delivery mechanism, options for titration, side effects, complexity of patient training and education, cost of therapy, availability of education materials, patient-specific factors such as age, education, and cultural considerations, and consideration of accepted treatment guidelines. No data will be collected about specific products or brands. The physician will also indicate his/her treatment and HbA1c goals for each enrolled patient at the baseline visit. Practice site characteristics (academic/non-academic, rural/urban, practice size, time dedicated to diabetes, and practice type [general medicine site/specialty site]) that may influence diabetes treatment practices will be enumerated as well.

### Data management

Practice site personnel will enter and submit patients’ clinical data electronically to a secure virtual data center. Electronic data entry forms feature automated quality checks to ensure that values are within range and meet quality standards. Self-reported data will be gathered using paper-and-pencil forms which are mailed to Quintiles for data entry into a database program featuring similar quality control.

### Primary outcome: insulin progression

Insulin progression is defined based on each patient’s insulin therapy regimen at the baseline visit (Table 4). If a patient begins the study on basal insulin, with or without any other non-insulin anti-diabetic medication, progression will be defined as the addition of prandial insulin, an increased frequency of insulin injections, change to an insulin mixture, or addition of a glucagon-like peptide 1 (GLP) medication or ≥1 oral anti-diabetic (OAD) medications. Alternatively, if a patient begins the study on an insulin regimen of basal insulin plus <3 prandial injections daily or is using insulin mixtures (both regimens may be used alone or with other non-insulin anti-diabetic medications), then progression is defined as an increased frequency of insulin injections, change to a basal-bolus regimen, or addition of a GLP-1 or ≥1 OAD medications. Importantly, this definition allows for the inclusion of T2DM patients who may be using insulin pumps, an emerging treatment approach among patients with T2DM. As MOSAIc will investigate the care patients receive in real-world settings, it is important to capture the spectrum of employed treatment strategies, even when these strategies may not reflect current established treatment guidelines. The progression date is the first visit date at which a patient is progressed or is referred to another health care provider for progression, as recorded in the medical record.

**Table 4:** Outcome definitions for insulin progression, based on initial insulin therapy

**Initial Insulin Therapy**	**Definition of Insulin Progression**^**a,b**^
Basal alone or in combination with any approved non-insulin anti-diabetic medications	Addition of prandial insulin (≥1 injection: basal plus or basal-bolus)
	Additional injections
	- QD to BID administration of NPH
	- Insulin glargine[Lantus]/Insulin levemir[Determir]
	- ILPS or insulin mixtures
	- Intermediate-acting NPH
	Mixtures
	- human/animal/analog premixed insulin (combination of short- and long-acting insulin in the same formulation)
	Addition of a GLP-1
	Addition of oral medications
Basal Insulin plus additional insulin therapy (less than a full basal-bolus regimen (basal + <3 prandial injections)) or in combination with any approved non-insulin anti-diabetic medications	Additional insulin injections (e.g., 1 to 2 injections, or 2 to 3 injections)
	Basal-bolus regimen
	Addition of a GLP-1
	Addition of oral medications
Insulin mixtures alone or in combination with any approved non-insulin anti-diabetic medications	Additional insulin injections (e.g., 1 to 2 injections, or 2 to 3 injections)
	Basal-bolus regimen
	Addition of a GLP-1
	Addition of oral medications

Abbreviations: *BID* = twice a day; *GLP-1* = glucagon-like peptide 1; *ILPS* = insulin lispro protamine suspension; *NPH* = neutral protamine Hagedorn; *QD* = every day.

^a^Referrals to a specialist (eg, endocrinologist) for progression of therapy will be captured.

^b^For the purpose of this study, progression will be defined as the *first* progression from initial therapy (as defined above).

### Statistical analysis

Multivariable regression modeling of the binary response variable (progressed/not progressed) will be conducted using a logistic main-effects regression model to examine the association between insulin progression and patient-, physician-, and healthcare environment-based factors. A generalized estimating equation (GEE) [24] will be used to estimate the parameters of the model and will take into account the clustering of patients within physicians and of physicians within clinical practice sites. Fifty risk factors assessed at baseline (Table 5) will be included as independent variables and will be evaluated for association with progression using False Discovery Rate p-values to maintain the type I error at 0.05 across the entire analysis. The 50 risk factors were selected based on expert subject matter knowledge and literature reviews conducted by the research team. Multiple imputation using Monte Carlo Markov Chain will be used in the modeling to address any missing data. Goodness-of-fit for the model will be examined using receiver operating characteristic analysis and the Hosmer-Lemeshow test.

**Table 5:** The 50 patient-, provider-, and health environment-specific variables included in the insulin progression prediction model


**Patient demographics**
1 Age
2 Gender
3 Education
4 Marriage Status
5 Work Status
**Patient clinical characteristics**
6 Duration of type II diabetes
7 Body mass index (BMI)*
8 Hemoglobin A1c (HbA1c)*
9 Low-density lipoprotein (LDL)*
10 High-density lipoprotein (HDL)*
11 Urine microalbumin to serum creatinine ratio*
12 Systolic blood pressure*
13 Number of days blood sugar was tested in the past week*
14 Daily units basal or intermediate insulin*
15 Daily units short-acting (regular) insulin*
**Patient medication history**
16 Metformin use †
17 Use of a sulfonurea †
18 Use of a glitazone †
19 Use of another oral antidiabetic drug †
20 Use of an ACE inhibitor ‡
21 Use of an ARB ‡
22 Use of a thiazide ‡
23 Use of a beta blocker ‡
**History of comorbidities§**
24 Hypertension
25 Cardiovascular disease^a^
26 Diabetes complications^b^
27 Hypoglycemia
**Patient self-care/healthy behavior measures at the baseline visit €**
28 Insulin-specific Adherence Questionnaire summary score
29 Current Fasting Practices
30 Current smoking use (any smoking during past 7 days)
31 Current Alcohol Use
32 Number of days on which patient exercised during the last 7 days
33 Insurance to pay for drugs
**Patient health care resource utilization in the 6 months prior to the baseline visit €**
34 Number of hospitalizations due to diabetes, including MI and ESRD
35 Number of diabetes-related visits to physician who treats patient’s diabetes
36 Number of visits to an auxiliary diabetes provider
**Physician-Related Variables∫**
37 Age
38 Gender
39 Practice affiliation (Academic versus non-academic)
40 Practice location (Urban versus rural)
41 Practice time dedicated to diabetes (patients/month)
42 Practice size
43 Practice type (primary care/internal medicine versus diabetologist/endocrinologist)
44 Prescribing choices and preferences Score
**Potentially Modifiable Variables**
45 Patient Relationship with Health Care Provider €
46 Physician’s target HbA1c level for this patient £
47 Experience with Insulin Treatment Questionnaire Score €
48 Diabetes Self Care Activities Survey Score €
49 Diabetes Knowledge Test Score €
50 Diabetes Distress Scale Score €

^a^ Definition includes presence of any of: transient ischemic attack, stroke, congestive heart failure, arrhythmia, coronary artery disease, myocardial infarction, peripheral arterial occlusive disease.

^b^ Definition includes presence of any of: retinopathy, nephropathy, neuropathy, hyperlipidemia, diabetic coma, hyperglycemic hypermolar nonketototic syndrome, amputation, arthritis, cancer/malignancy, depression, gastroparesis, diabetic foot, other skin infections, and oral infections, pyelonephritis, pneumonia, erectile dysfunction.

* Most recent values within the 6 months prior to the baseline visit, as recorded in the patient’s medical record.

† Ever use or concomitant use at the day prior to the baseline visit, either from self-report or the patient’s medical record.

‡ Concurrent use reported at the baseline visit via self-report or the patient’s medical record.

§ Determined via self-report at the baseline visit or from the patient’s medical record.

€ Via patient self-report at the baseline visit.

∫ Via physician self-report at the practice site enrollment.

£ Via physician self-report at the patient’s baseline visit.

### Sample size considerations

The sample size calculation was based on the inclusion of 50 independent variables in the primary multivariate regression model and 90% power to identify a predictor that impacts the likelihood of progression from 20% to 25% at a 0.001 level of significance (a type I error rate of 0.05 divided by 50 in a Bonferroni manner). We assumed a correlation of 0.5 among predictors. We estimate that 20% of patients will progress during the study and 20% will be lost to follow-up over the 24 month study period. Given these assumptions, 4,500 patients are needed to identify a predictor that impacts the likelihood of progression with a 5% change (from 20% to 25%) for a 1 standard deviation increase in the variable.

## Discussion

### Safety reporting

As MOSAIc is an observational study, there are no provisions for a data safety and monitoring board. Practice sites and physicians will continue to follow all applicable laws, regulations, and practices in their respective countries regarding reporting serious and non-serious events related to any anti-diabetic treatments, such as reporting events to government regulators and/or the local market authorization holder.

### Limitations

There are several important limitations to the proposed observational study. Simply observing and recording T2DM treatment patterns over the course of 24 months might produce a Hawthorne effect that influences patient and physician behaviors regarding insulin progression. In an attempt to quantify this effect, we measure patterns of T2DM treatment and care in the 6 months prior to the study so that a historical baseline is available. We only have access to a patient’s medical records as maintained by the physician who treats the patient’s diabetes and enrolls him in the MOSAIc study. If the patient sees other health care providers, the full extent of her comorbidities and prescription drug use may not be captured. However, patients will be able to self-report comorbidities and prescription drug use at their baseline visit, mitigating the loss of some information. Second, some treating physicians will refer their patients to another physician, often an endocrinologist or diabetologist, for the purposes of insulin progression and will note this on study forms. If these patients do not return to their original provider, the true progression status of these patients will be unknown. For the purposes of our analyses, we will include these patients and count them as meeting the definition of insulin progression, similar to an “Intention-to-Treat” analysis because the referring physician intended for the patient to be progressed. If these patients are not actually progressed by the new physician, this strategy will result in misclassification of their outcome. This approach may result in some bias in the parameter estimates, but we believe our strategy is conservative, more appropriate and potentially less biased than deleting these patients from the analyses. Finally, given sample size limitations and practicality, we consider 50 potential predictors of insulin progression in our primary prediction model. Additional predictors of progression might be identified and may be explored in sensitivity analyses.

### Funding and responsibilities

This study is funded by Eli Lilly and Company, Study F3Z-MC-B010. All data analyses and outcome assessments will be performed independent of the study sponsor by researchers at Brigham and Women’s Hospital and Harvard Medical School (BWH/HMS). Researchers at Eli Lilly and/or other organizations may contribute expertise to the study design and analytic plan; however, BWH/HMS researchers retain full and final control regarding the analytic plan, the decision to publish, and the final contents of manuscripts.

### Summary

The MOSAIc study is a multinational longitudinal observational cohort study among patients with T2DM that will specifically examine factors associated with insulin progression in real-world clinical practice. Extensive patient demographic, clinical, and psychosocial data as well as physician-specific and health care environment/practice-site data will be collected. The research team will capitalize on the breadth and depth of these data to explore and quantify predictors of insulin progression and to identify potential opportunities for health behavior intervention in order to improve T2DM treatment and clinical outcomes.

## Abbreviations

T2DM: Type 2 diabetes; MOSAIc: Multinational observational study assessing insulin use: understanding the challenges associated with progression of therapy; OAD: Oral antidiabetic medication; GLP: Glucagon-like peptide; GEE: Generalized estimating equation; BWH/HMS: Brigham and women’s hospital/harvard medical school.

## Competing interests

This study is funded by Eli Lilly and Company, Study F3Z-MC-B010.

Dr. Polinski is a consultant to Buccaneer Computer Systems and Service, Inc, a contractor for the Centers for Medicare and Medicaid Services. Drs. Polinski, Seeger and Shrank receive funding through a grant awarded to their employer, Brigham and Women’s Hospital, from Eli Lilly for the MOSAIc study. Drs. Curtis and Zagar are employees of and hold stock in Eli Lilly and Company. Dr. Shrank received research funding from CVS Caremark, Aetna, Express Scripts, Teva, the National Association of Chain Drug Store, and Eli Lilly and was a consultant for United Healthcare. Dr. Choudhry has no conflicts of interest to disclose. The authors retained independent and complete control over the design and implementation of the study as well as the analyses and writing of the manuscript.

## Authors’ contributions

All authors participated in the development of the study design and analytic plan. JP drafted the manuscript and BC, AZ, NC, JS, and WS reviewed and revised it critically for important intellectual content. All authors have given final approval of the version to be published.

## Pre-publication history

The pre-publication history for this paper can be accessed here:

http://www.biomedcentral.com/1472-6823/12/20/prepub

## Supplementary Material

Additional file 1 **Table S1.** Names of Ethics Review Boards granting approval to MOSAIc, by country. Click here for file
