# Supplementary material for: Rationale and design of the multinational observational study assessing insulin use: the MOSAIc study
Source: BMC Endocr Disord. 2012 Sep 21;12:20. doi: 10.1186/1472-6823-12-20 (PMC3545975; doi:10.1186/1472-6823-12-20)
Supplement: Additional file 1 — Table S1. Names of Ethics Review Boards granting approval to MOSAIc, by country. [file 1472-6823-12-20-S1.doc]

| **Country** | **Ethics Review Board** |
| --- | --- |
| USA | 1. Copernicus Group Institutional Review Board |
| Canada | 1. Health Research Ethics Authority |
|  | 1. Veritas Institutional Review Board |
|  | 1. Winnipeg Clinic |
|  | 1. College of Physicians and Surgeons of Alberta Research Ethics Board |
| Mexico | 1. COFEPRIS - Comision de Autorizacion Sanitaria |
|  | 1. Comite Independiente de Etica de Investigación y Bioseguridad del Bajio SC |
| Argentina | 1. Comite de Etica Independiente de Instituto de Investigaciones Clinicas Zarate |
|  | 1. Comite Independiente de Etica |
|  | 1. Comité de Ética de Protocolos de Investigación |
|  | 1. Comité de Ética en Investigación Hospital Sirio Libanés |
|  | 1. Comite de Docencia e Investigacion Clinica de CER San Juan |
|  | 1. COEIS-Consejo de Evaluación Ética de la Investigación en Salud |
|  | 1. Comité de Ética en Investigación |
| Brazil | 1. CONEP - Comissão Nacional de Ética em Pesquisa |
|  | 1. CEP em Seres Humanos do Centro Universitario Franciscano |
| China | 1. Ethics Committee of 306th Hospital of PLA |
|  | 1. Ethics Committee of General Hospital of PLA Beijing Region |
|  | 1. Ethics Committee of No.2 Hospital affiliated to Chongqing Medical University |
|  | 1. Ethics Committee of The 3rd Hospital of Hebei Medical University |
|  | 1. Ethics Committee of The 2nd Artilleryman General Hospital |
|  | 1. Ethics Committee of Country Hospital of Zhengding |
| Republic Of Korea | 1. Institutional Review Board of CHA Bundang Medical Center, CHA University |
|  | 1. Institutional Review Board of Jeju National University Hospital |
|  | 1. Institutional Review Board of The Catholic University of Korea Yeouido St.Mary's Hospital |
|  | 1. Institutional Review Board of Kangwon National University Hospital |
|  | 1. Institutional Review Board of Yeungnam University Hospital |
|  | 1. Institutional Review Board of Kwandong University College of Medicine Myongji Hospital |
|  | 1. Institutional Review Board of Chungnam National University Hospital |
|  | 1. Institutional Review Board of Keimyung University Dongsan Hospital |
| India | 1. Manipal University Ethics Committee |
|  | 1. Bangalore Diabetes Hospital Ethics Committee |
|  | 1. Institutional Ethics Committee, Monilek Hospital & Research Centre |
|  | 1. Human Welfare Ethical Committee for Human Sciences and Research |
|  | 1. Swasthya Kalyan Ethics Committee |
|  | 1. Institutional Ethics Committee, Apollo BGS Hospitals |
|  | 1. Ethics Committee, Apollo Hospitals |
|  | 1. MSRMC Ethical Review Board |
|  | 1. ACE Ethics |
|  | 1. Independent Ethics Committee, BYL Nair Hospital & TN Medical College |
|  | 1. Ethics Committee on Research on Human Subjects |
|  | 1. Ethics Committee of Diabetes Care and Research Centre |
|  | 1. Institutional Ethics Committee |
|  | 1. Astha Independent Ethics Committee |
|  | 1. Institutional Ethics Committee,N.K.P. Salve Institute of Medical Sciences & Lata Mangeshkar Hospital |
|  | 1. Aadhya Independent Ethics Committee |
|  | 1. Shashwat Ethics Committee, Shashwat Hospital & Research Centre |
|  | 1. Ethics Committee for Research |
|  | 1. Ethics Committee for Human Research, Star Hospitals |
|  | 1. Ethics review Board - Chowpatty Medical Centre |
|  | 1. Credo Independent Ethics Committee - Medi Heights Shreeji Health Care Hospital |
|  | 1. Ethics Committee Apollo Hospital Internationals Limited |
|  | 1. Ethics Committee of Biniwale Clinic (Independent Ethics Committee) |
|  | 1. Ethics Committee, Belgaum Diabetes Centre |
|  | 1. Institutional Ethical Review Board |
|  | 1. Ethics Committee, KEM Hospital Research Centre |
|  | 1. Diacon Hospital |
|  | 1. Clinical Trial Ethics Committee |
| Germany | 1. Aerztekammer Nordrhein |
|  | 1. Landesaerztekammer Rheinland-Pfalz |
|  | 1. Ethikkommission der Aerztekammer Sachsen-Anhalt |
|  | 1. Landesaeztekammer Hessen |
|  | 1. Saechsiche Landesaerztekammer |
|  | 1. Aerztekammer Hamburg |
|  | 1. Landesärztekammer Baden-Wuerttemberg |
| Russian Federation | 1. Ethical Council at the MoH of RF |
|  | 1. Local Ethics Committee under SHI of city of Moscow “City Clinical Hospital #4” |
|  | 1. Independent Ethics Committee under City Clinical Hospital # 11 |
|  | 1. Independent Ethics Committee under SIH City Clinical Hospital # 15 n.a. Filatov |
|  | 1. Local Ethics Committee under MHI "Vsevolozhsk Central Regional Hospital" |
|  | 1. Ethics Committee under SEIHPE Novosibirsk State Medical University |
|  | 1. Local Ethics Committee under Siberian State Medical University of Roszdrav |
|  | 1. Ethics Committee under Samara Regional Clinical Hospital n.a MI Kalinin |
|  | 1. Local Ethics Committee under SEIHPE "Rostov SMU of RosZdrav" |
|  | 1. Local Ethics Commitee at LLC International Medical Centre “SOGAZ” |
|  | 1. Ethics Committee at the RSMU n.a.N.I.Pirogov |
| Spain | 1. CEIC Fundació Gol i Gurina |
| United Kingdom | 1. NRES Committee North West – Preston |
| Italy | 1. Comitato Etico Locale per la Sperim. Clin. dei Medicinali dell'Az. Osp.ra Univ.ria Senese di Siena |
|  | 1. Comitato Etico per la Serimentazione Clinica dei Medicinali della AUSL 2 di Lucca |
|  | 1. Comitato Etico Indipendente dell'Osp Generale Regionale Francesco Miulli di Acquaviva delle Fonti |
|  | 1. Comitato di Bioetica dell'IRCCS INRCA di Ancona |
|  | 1. Comitato Etico dell'Azienda Ospedaliero-Universitaria Ospedali Riuniti di Foggia |
|  | 1. Comitato Etico Unico per la Provincia di Parma |
|  | 1. Comitato Etico Univ. Cattolica del Sacro Cuore Policlinico Universitario Agostino Gemelli |
|  | 1. Comitato Etico dell'Azienda Ospedaliera Antonio Cardarelli di Napoli |
| Turkey | 1. Istanbul University Cerrahpasa Ethics Committee |
|  | 1. Erciyes University Ethics Committee |
|  | 1. Turkish Ministry of Health |
| United Arab Emirates | 1. Ethics Committee of SKMC |
|  | 1. Ethics Committee of Al Ain Medical District |
|  | 1. Ethics Committee of Dubai Health Authority |
|  | 1. Ethics Committee of Al Qassimi Hospital |
| Saudi Arabia | 1. Ethics Committee, Al Noor Specialist Hospital |
|  | 1. Ethics Committee, Al Hada Military Hospital |
|  | 1. Ethics Committee of National Guards Health Affairs |
|  | 1. Ethics Committee, King Fahad Medical City |
|  | 1. Ethics Committee at King Abdulaziz University Hospital |
| Israel | 1. Sapir MC, Meir Hospital Ethics committee |
|  | 100. Assuta MC Ethics Committee |
|  | 101. Ziv MC Ethics Committee |
